# Does Telemedicine Promote Physical Activity?

**DOI:** 10.3390/life12030425

**Published:** 2022-03-15

**Authors:** Carolyn L. Rochester

**Affiliations:** 1 Section of Pulmonary, Critical Care and Sleep Medicine, Yale University School of Medicine, New Haven, CT 06520, USA; carolyn.rochester@yale.edu; Tel.: +1-203-785-4163; Fax: +1-203-785-3627; 2 VA Connecticut Healthcare System, West Haven, CT 06516, USA

**Keywords:** tele-rehabilitation, telemedicine, COPD, step count, accelerometer, physical activity, pulmonary rehabilitation

## Abstract

Exercise capacity and physical activity are different concepts: the former refers to what an individual is capable of performing, while the latter refers to what the individual does in daily life. Low levels of physical activity (PA), which are very common in individuals with COPD, are associated with poor health outcomes, including increased symptoms, a more rapid decline in lung function, increased health care utilization and increased mortality risk. Because of these pervasive negative outcomes, attempts have been made to increase physical activity in individuals with COPD, hoping that success in this area will mitigate the negative effects of inactivity. Based on its ability to increase exercise capacity and reduce dyspnea in COPD and other chronic respiratory diseases, pulmonary rehabilitation (PR) would be expected also increase physical activity in these patients. However, accessibility to pulmonary rehabilitation programs is problematic in some areas, and studies testing its effectiveness in this outcome area have had inconsistent results. Using telehealth interventions using technology to provide medical care conveniently over a distance would have the benefit of reaching a larger proportion of individuals with COPD. A systematic review of clinical trials testing telehealth to promote physical activity had mixed results and low-certainty evidence, resulting in the inability to recommend any single type of intervention. Thus, using telehealth interventions to promote physical activity for individuals with chronic respiratory diseases, while promising, remains an area where future investigations are needed to identify its optimal modalities and clarify its benefits.

## 1. Introduction

Exercise capacity and physical activity (PA) are different concepts. The former refers to what a person is inherently capable of doing, based on their anatomy and physiology, and the integrated interaction between the respiratory system, cardiovascular system and skeletal muscles and bones. Physical activity refers to what a person actually does in their daily life with regard to volitional use of their body to undertake activities of daily living, which may include various forms of exercise, and which leads to energy expenditure. The many dimensions of PA may be quantitated in several ways, including the type, frequency, intensity and total amount of the activity, and the energy expenditure it involves. Physical activity is an important determinant of health, which is necessary to function normally in daily life. At least 150 min of moderate intensity PA per week and muscle strengthening activity 2 or more days per week is recommended for adults [1,2]. Physical activity levels are impacted by genetic, physical, cultural, environmental, financial and motivational factors [3]. Low PA levels are associated with many adverse health outcomes, including obesity, cardiovascular disease, deconditioning, physical disability, type II diabetes, cancer, depression, cognitive impairment, osteoporosis, systemic inflammation, reduced quality of life (QOL), increased healthcare utilization and increased mortality [4,5,6,7,8,9]. Of note, sedentary behavior has been defined as waking behavior with energy expenditure < 1.5 metabolic equivalent task (METs) in sitting or reclining posture [7], and it specifically (distinct from PA per se) is an important determinant of health outcomes including mortality [7].

## 2. The Rationale for Physical Activity Promotion for Individuals with Chronic Obstructive Pulmonary Disease

Regular PA is also recommended for people with chronic obstructive pulmonary disease (COPD) [10]. However, people with COPD have low PA levels relative to healthy age-matched persons [11,12,13,14]. Among those with COPD, low PA levels are associated with worse pulmonary function, skeletal muscle dysfunction [15,16,17,18], sarcopenia [17,19], obesity [20] and lower exercise capacity [11,21]. Individuals requiring long-term oxygen therapy are particularly inactive [13,22]. Additional contributory factors affecting PA levels include comorbidities [23] (especially diastolic cardiac dysfunction) [24], depression [25], systemic inflammation [21], older age [13], having a physically active loved one [26], social support [27], time of year [28], living environment, motivation, self-efficacy and cultural influences [14]. As for healthy persons, low PA levels among those with COPD are associated with poor health outcomes [7], including worse symptoms [29], possible faster decline in lung function [7,30,31,32], increased hospitalization risk [33,34,35] and increased mortality [36]. As such, treatment interventions targeted to improving PA levels are desirable as a means to improve health outcomes for people with COPD. Physical activity is likely also important for individuals with other chronic respiratory diseases such as interstitial lung disease, pulmonary hypertension, cystic fibrosis (CF) and non-CF bronchiectasis, asthma and lung cancer and those preparing for or recovering from lung transplantation. However, this review will focus principally on COPD, since evidence regarding the impact of tele-rehabilitation on PA and other outcomes in non-COPD respiratory disorders is sparse. 

## 3. The Role and Limitations of Pulmonary Rehabilitation in Promoting Physical Activity

Pulmonary rehabilitation (PR) is a highly effective treatment that improves skeletal muscle function [15,37] and exercise capacity, reduces dyspnea and improves emotional function and QOL for people with COPD and other chronic respiratory diseases [38,39,40]. Despite its clear benefits regarding exercise capacity [40], its impact on PA levels is less consistent [14,41]. Although some studies have demonstrated increases in PA levels [42,43,44,45,46,47] and improved performance of activities of daily living [48] following participation in PR, other studies have not shown significant gains in PA outcomes [49,50,51,52]. Baseline exercise tolerance [53,54] and PR program duration [41] affect whether people improve their PA levels after participating in PR. Overall, changes in PA following PR are variable [41,55,56], and achieving gains in PA is challenging. An increase in PA of 600–1100 steps/day measured directly by accelerometry is proposed as the minimal important difference (MID) in PA after PR for patients with COPD [57]. The clinical importance of this change was supported by demonstration of reduced risk of hospitalization in those patients with >600 step improvement in PA [57]. The meaning of the proposed MID for other aspects of patients’ daily life is not yet fully clear. Activity coaching combined with PR confers greater benefit for increasing PA levels than PR alone [42]. 

The challenge of improving PA levels likely relates to the difference between what people “can do” versus what they “do do” [58]; although a threshold of exercise capacity is a necessary prerequisite to enable participation in PA, other factors such as motivation, confidence, self-efficacy, behavior change, social support and favorable environment are needed to foster and improve PA levels [38,59,60]. Moreover, there exist several health-system, geographic and patient-related barriers to PR [61]. Pulmonary rehabilitation programs are lacking in many geographic locations, especially rural areas [62,63] and low/middle income countries [64]. PR is, in general, under-funded and under-resourced. Even where present, existing programs accommodate a low number of individuals [65]. Suitable patients are often not referred to PR; even if referred, patients often lack sufficient information to decide whether they would like to participate [66]. Uptake and completion of PR is low [67,68,69]. Patient-related barriers such as lack of transportation, comorbidities, exacerbations, competing interests and travel distance all impact patients’ decision-making about and uptake of PR [67,68,69]. Given significant barriers to patients’ access to and uptake of traditional center-based PR, additional strategies are needed to expand PR access to a broader range of people who could benefit from it. 

Home-based PR (without telehealth) is one promising means of increasing patients’ access to and uptake of PR [70]. Home-based PR offers the potential benefits of greater time flexibility, lack of requirement for travel or costly exercise equipment, ability to foster exercise activity in patients’ own familiar environment and support from family and friends. For persons with COPD, home-based PR can result in significant short-term gains in exercise capacity (six minute walk test distance; 6MWD), dyspnea and quality of life [71], but the magnitude of gains may not be fully comparable to those made in traditional center-based PR for all outcomes [72]. Additionally, home-based PR may be less suitable for those with severe disease with severe gas exchange disturbances, multiple comorbidities, frailty and/or history of falls [70]. Further work is needed to identify optimal candidates for home-based PR.

## 4. Enhancing Pulmonary Rehabilitation Access and Uptake Using Telehealth

Delivery of PR via telehealth is an emerging novel strategy to expand people’s access to, as well as foster patients’ uptake and completion of, PR. Telehealth (telemedicine) refers to provision of health services to patients via electronic and telecommunication technologies, wherein advice, education, assessment, counseling, treatment and/or monitoring interventions are provided remotely. Tele-rehabilitation refers to delivery of rehabilitation services remotely via telecommunication technologies. This may include live remote interactions between patients and clinicians via videoconferencing or telephone, or asynchronous interactions via phone, tablet or computer apps. Tele-rehabilitation interventions may incorporate remotely supervised and/or unsupervised exercise training and/or education and/or physical activity coaching using remote monitoring conducted in the patient’s home or in a group setting in the community (e.g., hub and spoke model, with local healthcare staff monitoring patients directly while PR is delivered via videoconferencing from a central site) [73]. When administered in the home, patients typically perform exercise using minimal equipment (e.g., walking, stair climbing, sit to stand exercise, light strength exercises using household items or exercise stretch bands) and/or may use a treadmill or exercise bicycle if available. A broader range of physical exercise may be available in the community setting-type model if multimodality exercise equipment and staff supervision are available. 

Pulmonary rehabilitation delivered via telehealth (tele-PR) has the potential to overcome several barriers to traditional PR, including travel distance, lack of geographic access, patients’ reluctance to participate in group activity, inconvenient timing and other factors. Importantly, where studied to date, for those with COPD, tele-PR leads to several health benefits, including improvements in exercise capacity (6MWD) and QOL [63,74,75,76,77] generally comparable to those achieved in center-based PR, as well as decreased hospital readmission risk [78,79]. Tele-PR is also an attractive option for maintenance PR [80]. At least in the context of clinical trials, patients’ acceptance of tele-PR is high [81,82]. Tele-PR undertaken in patients’ homes has the potential to foster regular exercise in their usual daily environment and as such may promote increased participation in PA in addition to improving exercise capacity. 

Importantly, also, the recent and currently ongoing SARS-CoV2 pandemic has posed severe constraints on delivery of conventional, group- and center-based PR due to risks of disease transmission among and between patients and PR program staff. In most countries, PR programs were forced to close and suspend operations for extended periods of time. The pandemic has accelerated the broad-based use of telehealth for routine patient monitoring and outpatient care. Tele-PR has also emerged as an attractive model by which PR services can be delivered in patients’ homes during the pandemic while maintaining safety of patients and the PR program staff [83,84]. However, tele-PR has not yet been widely supported by or implemented in all health systems [85]. 

Given the adverse health outcomes associated with low PA levels, the need to improve PA levels among people with COPD, existing limitations on patients’ access to PR and the variable (and to date limited) impact of traditional PR on PA levels, and the desirability of tele-PR as a strategy by which PR can be delivered during a pandemic, work is ongoing to determine whether tele-PR or other interventions delivered remotely via telehealth can promote increased PA levels of people with COPD. A variety of interventions have been tested thus far, with mixed results [86]. Some studies have not found any benefit of supervised [76] or unsupervised [87] home-based tele-PR for improving PA levels. However, others have demonstrated encouraging results.

## 5. Telehealth Interventions to Promote Physical Activity in Individuals with COPD

A systematic review of randomized controlled trials (RCT) evaluating effects of telehealth with biofeedback on physical capacity, PA and dyspnea for people with COPD (9 studies, 982 patients; telehealth using variety of methods—phone calls, websites, mobile phones with/without education and/or exercise training) identified only three studies with PA as an outcome [86]. Two of these studies were excluded from analysis; composite results of the one trial with two groups analyzed (total 125 patients) showed a significant effect favoring telehealth for increasing PA levels (mean difference 64.7 min of unsupervised endurance exercise per week (walking, swimming, cycling); 95% CI 54.4–74.9). Tempering these positive results, a small, non-randomized study [87] evaluated PA levels one year after completion of a two-year tele-rehabilitation program for COPD patients. The initial program encouraged treadmill exercise at home while monitoring daily symptoms and training sessions on a webpage. While the initial study showed positive outcomes in several areas, one year after the intervention steps per day decreased from 3806 to 2817. Time spent on light PA also decreased over the interval, but moderate-to-vigorous activity was relatively maintained. 

Another small, non-randomized trial of tele-PR (28 sessions of strength and endurance exercise training using a satellite video platform with telemonitoring, video-patient assistance and phone calls) compared to historical controls who had undergone traditional center-based PR demonstrated comparable improvements in 6MWD dyspnea and QOL between groups, but greater increases in steps per day were noted following tele-PR compared with conventional PR (3412 vs. 1863 steps/day; *p* = 0.0002) [88]. However, 22% of the participants in the tele-PR group found the technology difficult to manage. 

An RCT of home-based tele-PR compared to usual medical care among 112 elderly patients with combined COPD and heart failure demonstrated significant improvements in 6MWD, as well as improvements in symptoms, QOL and longer median time to hospitalization or death in the intervention group; significant increases in self-reported PA levels were also seen as measured by the Physical Activity Scale for the Elderly (PASE) questionnaire, but unfortunately, no objective measure of PA levels was performed [89].

In an RCT evaluating impact of an internet-based telecoaching program, wherein US Veterans with COPD used a pedometer to track daily steps in combination with an internet-based program (which included step count feedback, individualized weekly goal setting, educational and motivational content and online social support) (intervention group) vs. pedometer use alone (controls), participants in the intervention group walked 779 more steps/day at 4 months compared to the controls (*p* = 0.005) [90]. However, a follow-up study showed that the increase in daily step count was not maintained at the 12-month time point [91]. 

Another RCT using an internet-based telecoaching program, wherein 109 US veterans with COPD (majority males, mean age 68.6, mean FEV_1_ 62.6 ± 21.6% predicted) were randomized to use a pedometer plus a website incorporating goal setting, feedback, disease-specific education and an online community forum vs. pedometer alone also demonstrated a significant increase in daily step count from baseline at 3 months (804 ± 356 steps/d; *p* = 0.02) among the intervention group relative to pedometer group [92]. In this trial, the difference in step counts between groups was evident within 3 weeks of program use, and a subgroup analysis found that the use of the website program in addition to a pedometer attenuated the decline in daily step count that occurred among participants between the summer and fall seasons. A further RCT using this pedometer plus an internet-based feedback and goal-setting intervention to foster PA (compared with pedometer alone) demonstrated an association between improvements in PA (steps/day) and improvements in exercise capacity (gains in 6MWD distance), improvement in lung function (FEV_1_ mL) and reduction in parameters of systemic inflammation (C-reactive protein and interleukin-6 levels) among responders to the intervention (i.e., those who had significant gains in steps/day; *n* = 62/99) [93]. 

Disappointingly, a multicenter RCT of a different PA intervention using biofeedback (using a smartphone APP, personalized activity goal setting, automated encouragement messages and instructions from a physiotherapist) in an effort to maintain PA levels after conventional PR failed to show improvement in or maintenance of PA from the intervention [94]. However, the intended PA and messaging from staff goals were suboptimal, and a high dropout rate was noted in this trial. 

Three additional recent RCT have demonstrated encouraging results regarding use of telehealth strategies to improve or maintain PA levels among individuals with COPD. The first is a multicenter study from six centers in Europe, involving a 12-week semi-automated multifaceted telecoaching intervention that combined a step-counter to provide patient feedback, a smartphone app that provided daily activity goals (which were revised weekly) and occasional telephone calls with the study staff. This study intervention led to significant increases (between-group differences) in steps per day (1469; 95% CI 971–1965; *p* ≤ 0.001), time spent in moderate intensity PA (10.4 min; 95% CI 6.1–14.7; *p* ≤ 0.001) and a modest increase in 6MWD (13.4 m; 95% CI 3.4–23.5; *p* < 0.01) as compared to the control group [95]. 

In the second, a 10-week RCT using a superiority design compared a supervised group-based tele-PR (60 min, three times weekly) program with a traditional center-based PR program (90 min twice weekly) among 134 participants with severe COPD (mean age 68 ± 9 years, mean FEV_1_ 33% ± 9%, baseline 6MWD (327 ± 103 M). This study showed no between-group differences for the change in 6MWD at end-intervention or at 22 weeks of follow up [96]. However, whereas participants in the traditional PR group had significant decreases in daily step count (assessed by triaxial accelerometer) over the study period from baseline to 22 weeks, the tele-PR group maintained similar step counts to their baseline levels. Thus, although the tele-PR intervention did not lead to significant gains in PA compared to baseline, it helped to prevent decline in PA over time. Additionally, tele-PR also led to greater improvements in COPD health status, anxiety and depression scores at the end of intervention (although not at 22 weeks), and there was a higher program completion rate in the tele-PR group (*n* = 57 vs. *n* = 43, *p* < 0.01). 

The third trial included 147 patients, comparing outcomes of three groups after a 2 month initial PR program: (1) a home-based maintenance tele-PR intervention; (2) a center-based outpatient maintenance PR program; and (3) usual care [79]. Maintenance tele-PR was comparable to center-based maintenance PR and was superior to usual care in maintaining PA levels over 12 months. 

Finally, a recent Cochrane Review of interventions to promote PA among people with COPD [97] found that exercise training, PA counseling and COPD medications can improve PA levels. However, based on the low-certainty evidence available to date, the small number of studies using variable intervention designs and conducted across varying health systems and the demonstration of mixed results, no single type of intervention could be recommended. Thus, firm conclusions on the effectiveness of the interventions and the optimal model(s) or components remain unclear.

## 6. Limitations of Evidence Regarding Telehealth Interventions to Promote Physical Activity

Although in several of the trials discussed above, the gain in step count following tele-PR (where reported) was greater than the proposed MID of 600 steps/day [57], one key limitation of the existing literature regarding the impact of tele-PR on PA levels among individuals with COPD is that the number of steps per day documents only one of several dimensions of PA [7,98]. Other aspects of PA not captured by steps per day include activity type, total amount, intensity, time of day or sedentary behavior. In addition to the measurement of steps per day, it would be of critical interest to learn whether tele-PR can affect PA assessed by other outcome measures such as the International Physical Activity Questionnaire (IPAQ) [99] and/or intensity of activity and reduction in sedentary time assessed by accelerometry. For example, although there is potential risk of recall bias, the IPAQ form asks not only about walking but also about moderate- and vigorous-intensity activities (time and frequency of events) and sedentarism; patient responses are then converted to MET levels. Characterization of a broader range of dimensions of PA using IPAQ and/or accelerometry (or other outcomes tools) for individuals with COPD and other respiratory diseases after tele-PR is an important area for future research. Indeed, improvements in IPAQ scores have been noted following participation in conventional PR among persons with non-CF bronchiectasis [100] and idiopathic pulmonary fibrosis [101] and after tele-cardiac rehabilitation [102]. 

An additional important limitation of the existing evidence regarding tele-PR for people with COPD is that recruitment bias may have affected the findings of the studies. Patients selected by rigorous trial eligibility criteria and/or who are highly motivated to participate in clinical trials may potentially exhibit greater benefits from tele-PR than those who have greater lung disease severity, higher burden of medical comorbidity and/or less general health literacy and/or telehealth/technology skills. Additional pragmatic trials of tele-PR are needed incorporating individuals with a range of these characteristics to more comprehensively assess the real-world benefits of tele-PR. Likewise, the long-term widespread impact of tele-PR remains to be proven, and as for conventional center-based PR, the best means of maintaining the benefits of tele-PR is currently unknown. 

Lastly, while PR is beneficial for many forms of chronic respiratory disease [38], very few data exist to date regarding tele-PR for non-COPD respiratory disorders. In the 2021 Cochrane Review on tele-rehabilitation for chronic respiratory disease [74], only 1% of the participants (out of 1904 total) in the 15 studies included (three controlled trials) had non-COPD respiratory disease. Pilot studies of tele-PR among people with non-COPD respiratory disorders are, however, emerging. A small uncontrolled pilot trial of home-based tele-PR using a fitness app and self-selected activity among patients with severe CF awaiting lung transplant demonstrated lower decline in 6MWD among program completers vs. non-completers; adherence to the tele-PR program was higher than historically noted for center-based PR, and no adverse events were noted [103]. Physical activity was not assessed in this trial. A randomized trial of 3 months of tele-PR among 29 individuals with IPF with remotely delivered physical therapy sessions demonstrated better maintenance of the 6MWD over 6 months in the tele-PR group compared to the control group. No significant difference was found between groups for pedometer-measured steps per day [104]. Adherence to the intervention was 64% over the first 3 months; no adverse events were recorded. Taken together, given the paucity of data, it is not currently possible to draw conclusions regarding the safety and efficacy of tele-PR for non-COPD respiratory disorders. This is another area that merits further research. 

## 7. Conclusions

The field of using telehealth to improve PA of people with COPD and other chronic respiratory diseases is in its infancy. While some studies suggest promising results, not all studies have shown positive findings. The mixed findings of existing studies likely relate to variable study design, type and duration of tele-PR interventions provided and patient populations included. Importantly, not all patients require the same treatment interventions. For example, while some need exercise training to improve exercise capacity such that they have greater potential to engage in PA, others with relatively preserved exercise capacity may principally require strategies to improve motivation and behavior change to incorporate more PA in their daily lives. Others may need treatment directed towards anxiety, depression, cognitive impairment, other aspects of psychosocial support and/or other comorbidities to improve their daily function and PA levels. Some patients may benefit most from (and/or prefer) center-based or home-based PR without telehealth. Others may benefit most from and/or prefer to participate in tele-PR. Hybrid models of PR, incorporating a combination of directly supervised (center- or home-based) and tele-PR exercise and/or education and/or behavior change and PA coaching may be optimal for some individuals. As considered recently in the ATS Workshop Report on “Defining Modern Pulmonary Rehabilitation”, a menu-based approach based on a comprehensive multifaceted PR assessment may prove useful for assessing individual patients’ needs, treatment goals, priorities and preferences and in turn guide the choice of optimal PR program setting and content [70]. Sequential program-type models, in which patients begin in one PR program setting (e.g., center-based supervised PR) and transition over time to a minimally supervised or unsupervised (e.g., tele-PR) model at home, may be optimal for some patients [70]. 

The existing evidence regarding tele-PR does not yet allow any firm conclusions to be drawn regarding what type of tele-PR treatment should be provided, what telehealth tools should be used, or what patient characteristics define the optimal candidates to receive tele-PR, particularly in regard to achieving improvements in PA levels. Further research is needed to determine whether and to what extent various types of telehealth interventions (including video- or APP-based coaching (either alone or in combination with supervised or unsupervised exercise training), individual and/or group-based remotely delivered tele-PR or other novel models) can improve PA levels in the short and/or long terms, as well as which profiles of patients benefit most from varying types of interventions. Importantly, as emphasized by Burge and colleagues in their recent Cochrane Review [97], the best means of improving PA levels in the long term, which is likely required to achieve long-term health benefits, remains unknown at present. Moreover, while more clarity is certainly needed regarding the ability of telehealth to foster PA among people with COPD, its ability to promote PA among those with non-COPD respiratory disorders is equally important. For all people with chronic respiratory diseases, the potential relationship between improved PA levels afforded by telehealth interventions and other health outcomes is an essential area of future study. 

Ultimately, the precise type, content, setting and duration of PR (including tele-PR) will depend in significant part on the availability of services present in any given geographic region and health system. Further research relating to benefits, strategies and cost-effectiveness of tele-PR should help to foster health-system support for more widespread implementation of tele-PR. Engagement of patients and shared decision-making between patients and clinicians regarding choices pertaining to PR and regarding advocacy for PR availability are also of key importance. 

## Data Availability

This review article did not report any primary data. It summarizes data reported in studies published previously.
